# Incidence and characterization of spontaneous pituitary neuroendocrine tumors in aged spontaneously hypertensive rats

**DOI:** 10.1038/s41598-025-26871-8

**Published:** 2025-11-07

**Authors:** Anna C. J. Kalisvaart, Frank K. H. van Landeghem, Craig W. Wilkinson, Frederick Colbourne

**Affiliations:** 1 Department of Psychology, University of Alberta, Edmonton, Canada; 2 Neuroscience and Mental Health Institute, University of Alberta, Edmonton, Canada; 3 Burke Neurological Institute, Weill Cornell Medicine, White Plains, USA; 4 Department of Laboratory Medicine and Pathology, University of Alberta & University of Alberta Hospital, Edmonton, Canada; 5 Research Ethics Office, University of Alberta, Edmonton, Canada; 6 Women and Children’s Health Research Institute, University of Alberta, Edmonton, Canada

**Keywords:** Hypertension, Pituitary adenoma, Pituitary neuroendocrine tumor, PitNET, Pituitary NET, Spontaneously hypertensive rat, Intracranial tumor, Brain tumor, CNS cancer, Stroke, Cancer in the nervous system, Stroke

## Abstract

Primary intracranial tumors make up ~ 2% of all cancer cases but contribute disproportionately to cancer-related morbidity and mortality. While spontaneous preclinical intracranial tumor models offer valuable insights into the mechanisms underlying primary tumor formation and progression, few have been identified to date. As such, tracking and characterizing spontaneous tumor development in aging laboratory animals represents an opportunity to advance preclinical research, while informing best practices for animal welfare. Here, we investigated intracranial tumor incidence and origin in a cohort of spontaneously hypertensive rats aged 20–24 months (SHRs; *N* = 60), initially intended for preclinical stroke experiments. Upon gross inspection, we identified large intracranial tumors in 30% of aged SHRs. Using histological, immunohistological, and ultrastructural methods, we found that ~ 89% of these tumors were pituitary neuroendocrine tumors (NETs), with an average tumor diameter of 6.7 ± 1.22 mm (mean ± 95% confidence interval). These pituitary NETs displayed rapid growth, invasive behavior, and aggressive proliferation, as indicated by Ki67 staining. Our results reveal a predisposition in aged SHRs to develop aggressive pituitary NETs, providing a diagnostic and pathological foundation for future mechanistic and translational studies, while emphasizing the need for enhanced surveillance and care in aging laboratory animal populations.

**Supplementary Information:**

The online version contains supplementary material available at 10.1038/s41598-025-26871-8.

## Introduction

Worldwide, the annual incidence of intracranial tumors is approximately 20 per 100,000 people^1^. While primary intracranial tumor diagnoses only make up approximately 2% of all cancer cases, they disproportionately account for cancer-related morbidity and mortality^2^. Despite ongoing advances in neuroimaging and treatment, outcomes for patients with intracranial tumors remain suboptimal, and causal factors, aside from heritable disorders, are poorly understood^2,3^. As such, there is much interest in improving models of primary intracranial tumor development and progression in order to enhance strategies for prevention, detection, and treatment.

Animal models of spontaneous brain tumor formation offer several potential translational advantages. The lack of external manipulation in spontaneous tumor models offers greater physiological relevance, and may better capture the complexity of the tumor microenvironment and tumor evolution compared to genetic knock-out or knock-in models, chemical or radiation induced mutagenesis, or xenografts^4,5^. Non-human mammals develop spontaneous intracranial tumors at approximately the same rate as humans (~ 0.02–0.5%)^6^, with the exception of rats^7^. Estimates of lifetime intracranial tumor incidence in common laboratory rodent strains range from 0.5 to 5% depending on the survival period^8–14^ and diagnostic methodology (microscopic versus gross tissue evaluation)^13^. Some report higher rates with advanced age (e.g., 2 + years), particularly in Wistar rat strains^15–19^. Low incidence rates and lack of predictability generally limit the use of rodent spontaneous brain tumor models^4,5^, although valuable insights have been generated from serendipitous findings in the past, pointing to the potential translational value of such cases. For instance, a germline mutation discovered in Sprague-Dawley rats mirroring human multiple endocrine neoplasia (MEN) syndrome resulted in the discovery of a new human MEN variant, MEN4^20,21^. The “MENX” rat model, which harbors this mutation, is now used for preclinical study of MEN4^20–22^. Given the potential for such findings, tracking spontaneous tumor formation in various rodent strains across the lifespan holds promise for advancing clinical research and therapeutic development.

Studying and tracking spontaneous tumor formation in laboratory rats as they age is also ethically important, particularly in light of the growing emphasis on the use of aged and comorbid animals in different translational research disciplines, such as stroke^23–25^ and cancer^26–28^. As research shifts towards more representative models of human disease, including age-related conditions and comorbidities, it is important to account for the natural disease processes that occur in laboratory animals, and understand how these may affect experimental results. Close monitoring of tumor formation and other age-related conditions in laboratory animals is also needed to ensure animal welfare, and enhanced reporting of this data will help improve detection and management of age-related diseases in these populations, particularly in the case of strain-specific vulnerabilities. Early detection of spontaneous intracranial tumors could allow for better management of potential health complications, minimizing suffering and maintaining humane research conditions. Such an approach supports the responsible use of research animals while maintaining compliance with ethical standards for animal care.

Therefore, in this study, we investigated the formation of primary intracranial tumors in a cohort of aged spontaneously hypertensive rats (SHRs), a commonly used animal model in cardiovascular research^29^. With age (e.g., 20–24 months), our SHR colony naturally began to develop large intracranial tumors at a high rate, the incidence and origin of which has not been previously documented, at least to our knowledge. Here, we tracked the incidence of these primary intracranial tumors, and used a combination of histological, immunohistological, and ultrastructural methods to characterize their origin. Given the unexpected nature of these findings, our goal was to document their frequency and pathology in detail, and to provide a rigorous descriptive framework that could inform future mechanistic investigation.

## Methods

### Subjects

All experiments were approved by a University of Alberta Animal Care and Use Committee (Protocol #960) and were performed in accordance with the relevant guidelines and regulations. All work was conducted and reported in concordance with the ARRIVE guidelines^30^. A cohort of male SHRs (*N*= 60; strain: SHR/NCrl), a well-established inbred strain derived from Wistar-Kyoto rats^31^, was obtained from Charles River (Saint Constant, QC) and aged out to 20–24 months for other preclinical stroke experiments^32^. Animals were aged in-laboratory beginning from 2 to 3 months old and were kept in environmentally enriched social housing (e.g., extra chewing and nesting material, alternating toys) throughout the aging process, though some animals were briefly singly housed for up to 72 h prior to euthanasia as part of their intended use in other experiments^32^. All rats had *ad libitum* access to food and water. The laboratory environment was temperature- and humidity-controlled, with a lights-on cycle of 7:00 am to 7:00 pm; all work was conducted over light periods.

### Experimental protocol

**Fig. 1:**
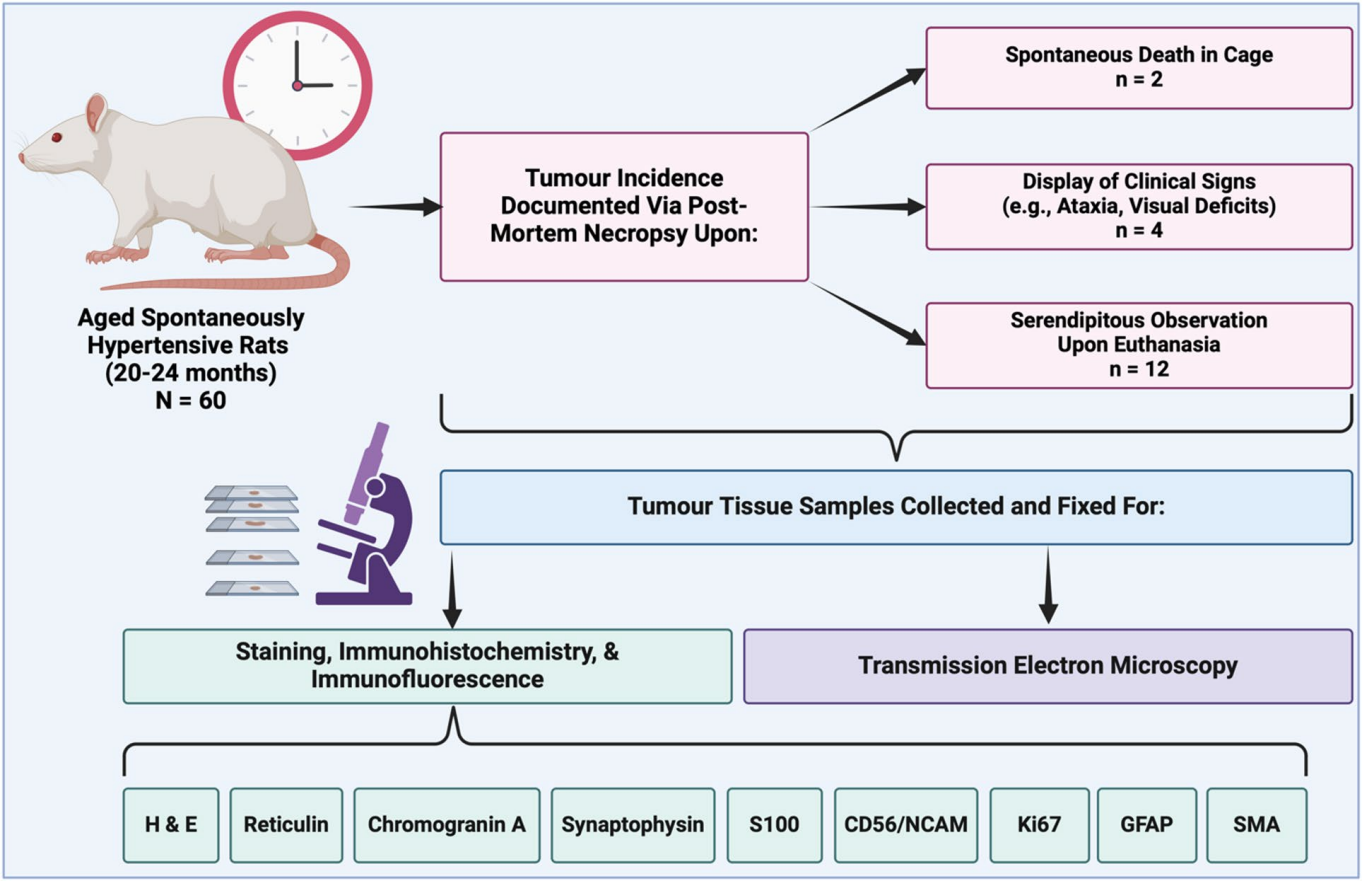
Primary intracranial tumor incidence was documented longitudinally in a cohort of sixty SHRs aged out to 20–24 months, originally intended for preclinical stroke experiments with an experimental endpoint of either 24–72 h following stroke induction (collagenase intracerebral hemorrhage) or sham control procedure (e.g., Kalisvaart et al., in press^33^; Wilkinson et al. 2023^32^). Tumors were found in both stroke animals (*n* = 8) and naïve/sham animals (*n* = 10). Intracranial tumor incidence was documented following either: (1) spontaneous death in cage; (2) clinical signs (e.g., ataxia, ocular irritation, weight loss, seizures), which met criteria for premature euthanasia (e.g., weight loss non-responsive to treatment, observable seizures, mobility issues affecting feeding/drinking behavior) prior to experimental endpoint in 50% of cases; or (3) upon serendipitous observation following planned euthanasia (e.g., no obvious clinical signs of brain tumors prior to experimental endpoint). Tissue samples were taken for assessment in consult with a neuropathologist (routing staining, immunohistochemistry, immunofluorescence, transmission electron microscopy). Intracranial sellar tumors were assessed via a series via a series of targets, based on their location and assessment of hematoxylin & eosin (H&E) sections. These included reticulin, chromogranin A, synaptophysin, S100, CD56/neural cell adhesion molecule (NCAM), Ki67, glial fibrillary acidic protein (GFAP), and smooth muscle actin (SMA). *Created in BioRender. Kalisvaart*,* A. (2025)*. https://BioRender.com/*c74d283*.

Within our cohort of sixty aged SHRs, primary intracranial tumor incidence was documented during post-mortem necropsy under the following circumstances: (a) spontaneous death in cage, (b) observation of clinical signs (e.g., ataxia, loss of balance/coordination, visual deficits) that met criteria for premature euthanasia prior to any experimental manipulation, or (c) unforeseen discovery following euthanasia with no obvious clinical signs (Fig. 1). When possible, tumor length was measured along both rostral-caudal and medial-lateral axes, and the average tumor diameter was calculated based on these values. Brain and tumor tissue were then extracted and collected for further processing, including assessment of tumor diameter, histology, immunohistochemistry, immunofluorescent staining, and transmission electron microscopy (TEM).

### Diagnostic histology and ultrastructural evaluation

All diagnostic histological and ultrastructural assessments were both recommended and reviewed with a neuropathologist (F.K.H.L).

#### Routine staining

A representative subset of sellar region tumor samples (*n* = 6) were fixed either via transcardial perfusion using 0.9% saline and 10% neutral-buffered formalin (#SF10020, Thermo Fisher Scientific Inc., Waltham, MA) after animals were euthanized via sodium pentobarbital (100 mg/kg IP, Bimeda, Cambridge, ON). Tumor samples were subsequently paraffin embedded and sectioned at 5 μm by the University of Alberta Laboratory Medicine and Pathology Core. Several cerebellar samples collected from non-tumor aged SHRs were embedded to act as control tissue (*n* = 2). Once collected, one set of sections were routinely stained with hematoxylin and eosin (H&E; #3801654, Leica Biosystems, Nussloch, Germany**)**, while another was stained with reticulin (#3804040802, Leica Biosystems); a third set of sections was set aside for subsequent immunofluorescent staining (see Sect. 5.2.4.3 below). Slides were imaged at 40x magnification using a Nikon Eclipse E600 (Nikon Instruments, Melville, NY), equipped with a Nikon DS-Fi3 camera (Nikon Instruments, Melville, NY), and NIS Elements F software (v. 5. 21; Nikon Instruments, Melville, NY).

#### Immunohistochemistry

Paraffin embedded tumor and control samples were sent to the Penn Vet Comparative Pathology Core, at the University of Pennsylvania. For immunohistochemistry, 4 μm thick paraffin sections of tumor and control tissue samples were mounted on ProbeOn™ slides (#1518851, Thermo Fisher Scientific Inc.). As reported by the facility, the immunostaining procedure was performed using a Leica BOND RXm automated platform combined with the Bond Polymer Refine Detection kit (#DS9800, Leica Biosystems). Briefly, after dewaxing and rehydration, sections were pretreated with the epitope retrieval BOND ER2 high pH buffer (#AR9640, Leica Biosystems) for 20 min at 98 °C. Endogenous peroxidase was inactivated with 3% H_2_O_2_ for 10 min at room temperature (RT). Nonspecific tissue-antibody interactions were blocked with Leica Biosystems PowerVision Super Blocking solution (#PV6122) for 30 min at RT. The same blocking solution also served as diluent for the primary antibodies. Rabbit monoclonal primary antibodies against glial fibrillary acidic protein (GFAP; #80788, Cell Signalling Technologies [CST], Danvers, MA,), smooth muscle actin (SMA; #19245, CST), neural cell adhesion molecule (NCAM/CD56; #99746, CST), synaptophysin (#36406, CST), S100 (#Z0311, Agilent-DAKO, Santa Clara, CA), and chromogranin A (#ab283265, Abcam, Cambridge, UK) were used at a concentration of 1/800, 1/200, 1/200, 1/50, 1/100, and 1/500 respectively. Antibody solutions were incubated on the sections for 45 min at RT. A biotin-free polymeric immunohistochemistry detection system consisting of horseradish peroxidase (HRP) conjugated goat anti-rabbit (#DS9800, Leica Biosystems) was then applied for 25 min at RT. Immunoreactivity was revealed with the diaminobenzidine (DAB) chromogen reaction. Slides were finally counterstained in hematoxylin, dehydrated in an ethanol series, cleared in xylene, and permanently mounted with a resinous mounting medium and the ClearVue™ coverslipper (Thermo Fisher Scientific Inc.). Control brain tissue sections were used as the positive control. Negative controls were obtained either by omission of the primary antibody or replacement with an irrelevant isotype-matched monoclonal antibody. Slides were then imaged at 40x magnification using either the Leica Aperio VERSA 200 digital pathology scanner or a Nikon Eclipse E600 microscope, equipped with a Nikon DS-Fi3 camera and NIS Elements F software, as described above.

#### Immunofluorescence

One set of the 5 μm paraffin embedded slides (tumor and control brain tissue) were used for immunofluorescent Ki67 staining, with the addition of rodent liver tissue sections to act as a positive proliferative control. After dewaxing and rehydration, slides underwent heat-induced epitope retrieval by microwaving slides in citrate buffer (#C999, MilliporeSigma) at 100% power for 1 min, and 20% power for 20 min in a 700-watt microwave. Slides were washed with distilled water, and a hydrophobic barrier was drawn around tissue sections using a PAP pen (#Z377821, MilliporeSigma). Slides were placed in a humidified chamber and incubated with a blocking buffer of 5% bovine serum albumin (BSA; #A7030, MilliporeSigma**)** and 0.3% Triton™ (#X100, MilliporeSigma) in phosphate buffered saline (PBS; #P4417, Millipore Sigma**)** for 1 h at RT. A rabbit monoclonal primary antibody against Ki67 (#MA5-14520, Thermo Fisher Scientific Inc.) was used at a concentration of 1/500 in 1% BSA + 0.3% Triton in PBS, and slides were incubated at 4 °C overnight. Slides were then washed with PBS + 0.05% Tween^®^ (#524653, MilliporeSigma) and incubated in a humidified chamber for 1 h at RT with a donkey anti-rabbit IgG (H + L) CF^®^594 secondary antibody (#SAB4600099, MilliporeSigma), diluted 1/1000 in 1% BSA + 0.3% Triton in PBS. Slides were counterstained and cover slipped using Fluoroshield™ with DAPI (#F6057, MilliporeSigma), then sealed with clear nail polish.

To determine the Ki67 proliferative index for each tumor sample, ~ 1500 tumor cells were evaluated across five images taken at 40x magnification, using the CellSens Dimensions software (v.3.2, Olympus Life Sciences, Japan) and an Olympus BX51 microscope (Olympus Life Sciences, Japan), outfitted with an Olympus DP74 digital camera and a motorized XYZ stage controller (Marzhauser Wetzlar, Germany). Out of the five images, three were taken at the tumor margins (more likely to contain proliferating cells), and two were taken within the tumor core, as is recommended for reproducibility^34,35^. The index value represents the average percentage of Ki67 + stained cells relative to the total number of DAPI stained cells across images. Cells were considered Ki67 + only if there was clear evidence of nuclear localization^34,36^.

#### Transmission electron microscopy

Several tumor samples were collected following fixation via transcardial perfusion with 0.9% saline followed by a modified Karnovsky’s fixative, as described previously^37^. Tumor samples were trimmed, processed, and embedded in resin, followed by ultra-thin sectioning at 90 nm. Sections were mounted on grids, stained with lead citrate and uranyl acetate, and imaged using a Philips-FEI Morgagni 268 80 kV transmission electron microscope (FEI Company, Hillsboro, OR) outfitted with a Gatan CCD camera (Gatan Inc., Pleasanton, CA) at various magnifications.

### Statistical comparisons

All data were graphed using GraphPad Prism (v.10.3.1, GraphPad Software Inc., La Jolla, CA), and presented as mean ± 95% confidence interval (CI). Statistics reported in text are mean ± 95% CI, unless otherwise specified.

## Results

### Tumor incidence & gross morphology

**Fig. 2:**
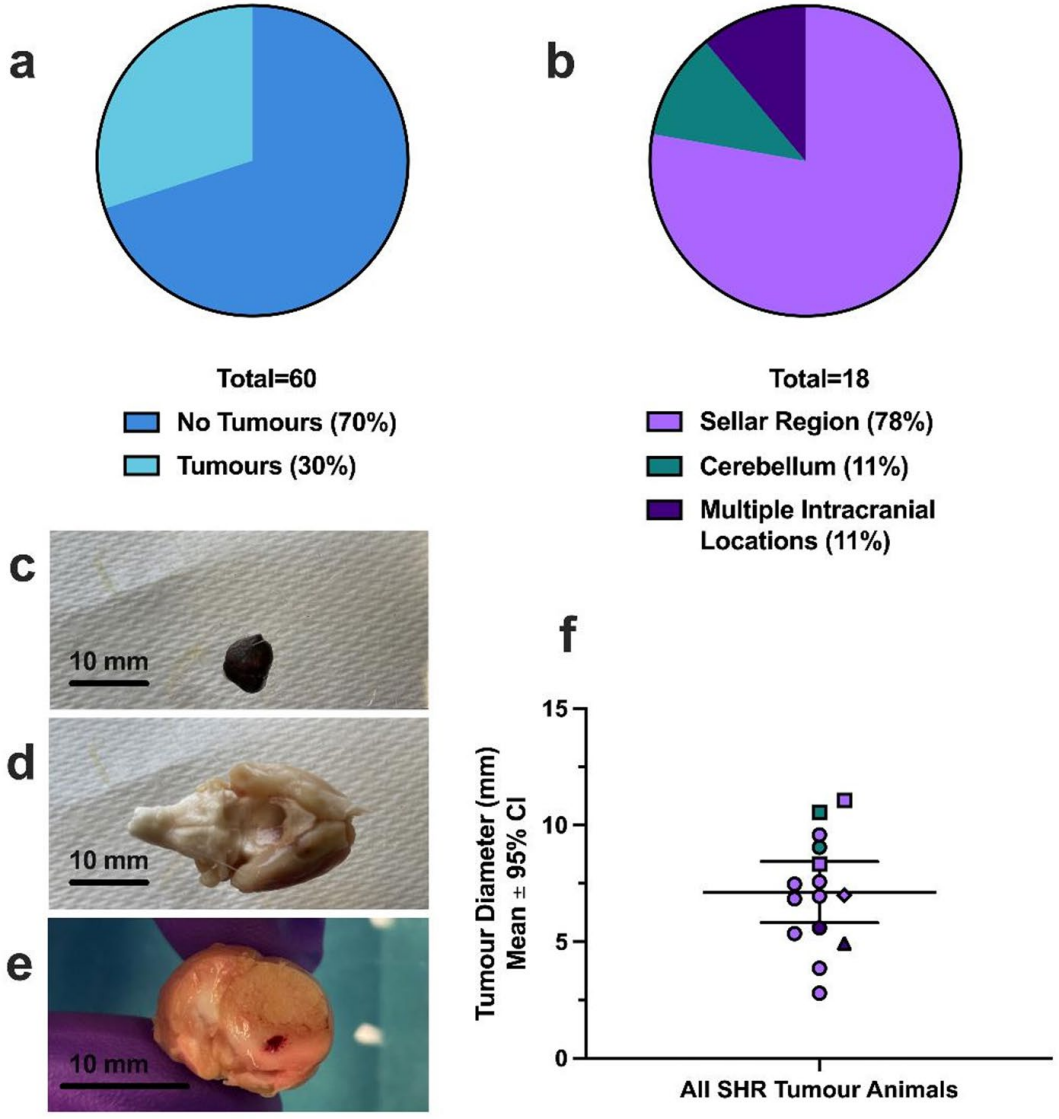
Intracranial tumor incidence and gross morphology in a cohort of sixty aged SHRs. **(a)** 30% of aged SHRs developed tumors, at an average age of 648 days old. **(b)** Of these tumors, 78% were in the sellar region beneath the pituitary and cerebellum, 11% were located on the superior aspect of the cerebellum, and 11% were sellar region tumors with evident brain infiltration into cortical and subcortical structures. Representative images of an intracranial sellar tumor with a diameter of 7.57 mm **(c)**, and the brain it was detached from **(d)**. In some cases, notable subcortical and cortical brain infiltration occurred **(e)**, originating from a sellar region tumor (located posterior to coronal section shown). This animal was originally allocated for experimental ultrastructural evaluation following collagenase intracerebral hemorrhage (e.g., resulting in hematoma pictured) as part of a preclinical stroke study. **(f)** Diameter measurements of extracted tumors across all intracranial locations. In rodents where measurements were possible, tumor radius averaged 6.98 ± 1.59 mm in those with sellar region tumors, 9.80 ± 9.57 mm in animals with cerebellar tumors, and 5.57 ± 10.10 mm in animals with sellar region tumors and notable brain infiltration. Square symbols denote animals who were euthanized following clinical signs, triangle symbols denote animals who were found dead in cage, and diamond symbols denote animals were found dead in cage with a history of clinical signs. Data points are colourized by tumor location, as per panel 2b.

In our cohort of sixty male aged SHRs, 18 out of 60 developed intracranial tumors (30%) as noted at an average of ~ 648 days old and a range of 178 days, or 1.8 years of age and a range of 5.7 months (Fig. 2a). Of these tumors, 78% were located on the ventral aspect of the brain at the sellar region (Fig. 2b, c, d), 11% were documented in the sellar region but with evidence of substantial cortical and subcortical brain infiltration (e.g., multiple intracranial locations; Fig. 2b and e), and 11% were located on the superior aspect of the cerebellum, with no obvious sellar tumors, at least upon gross inspection (one of which was large enough to result in notable distortion of supratentorial structures; Fig. 2b). In those that only had sellar region tumors and were formally evaluated for gross tumor morphology upon euthanasia (11 out of 14 animals), 1 animal exhibited a primarily intrasellar tumor, while the remaining 10 had both notable intrasellar and suprasellar growth that caused appreciable distortion of proximal structures, such as overlying brain tissue (Fig. 2d). Sellar tumors were not easily extractable, with possible parasellar infiltration of surrounding structures, such as surrounding dural membranes and the basisphenoid bone (as evidenced by gross evaluation of tumor margins). In all cases, we did not observe obvious metastases outside of the cranium upon post-mortem necropsy. Although cerebellar tumors did not appear to have a sellar origin upon gross morphological inspection, it remains possible that smaller sellar abnormalities went undetected.

Out of the 18 animals found with detectable intracranial tumors, 4 developed noticeable clinical signs, such as ataxia, weight loss, ocular irritation, and/or seizures (Fig. 2f), meeting criteria for premature euthanasia prior to experimental endpoint (e.g., 24–72 h post-stroke or sham procedure) in 50% of cases (Supplemental Material 1). Two were found dead in their cage, one of which also had prior clinical signs noted (Fig. 2f; Supplemental Material 1). Tumors in the remaining 12 animals were discovered after euthanasia at their planned experimental endpoint, with no prior clinical signs (Fig. 2f; Supplemental Material 1). These animals appeared normal to both animal care staff and investigators (e.g., no formal behavioural or neurological testing was done to identify them). Across all intracranial locations, the average tumor diameter was 7.1 ± 1.3 mm (Fig. 2f); assuming a roughly spherical tumor shape, this constitutes a volume of ~ 248.1 ± 116.8 mm^3^, or ~ 10–20% of total brain volume in aged SHRs^32,38^. The average tumor diameter was 7.0 ± 1.6 mm in animals with sellar region tumors, and 9.8 ± 9.6 mm in animals with tumors located on the superior aspect of the cerebellum. In animals who had sellar tumors with notable brain infiltration, sellar tumor diameter averaged 5.6 ± 10.1 mm.

### Diagnostic histology & transmission electron microscopy

#### Routine staining

**Fig. 3:**
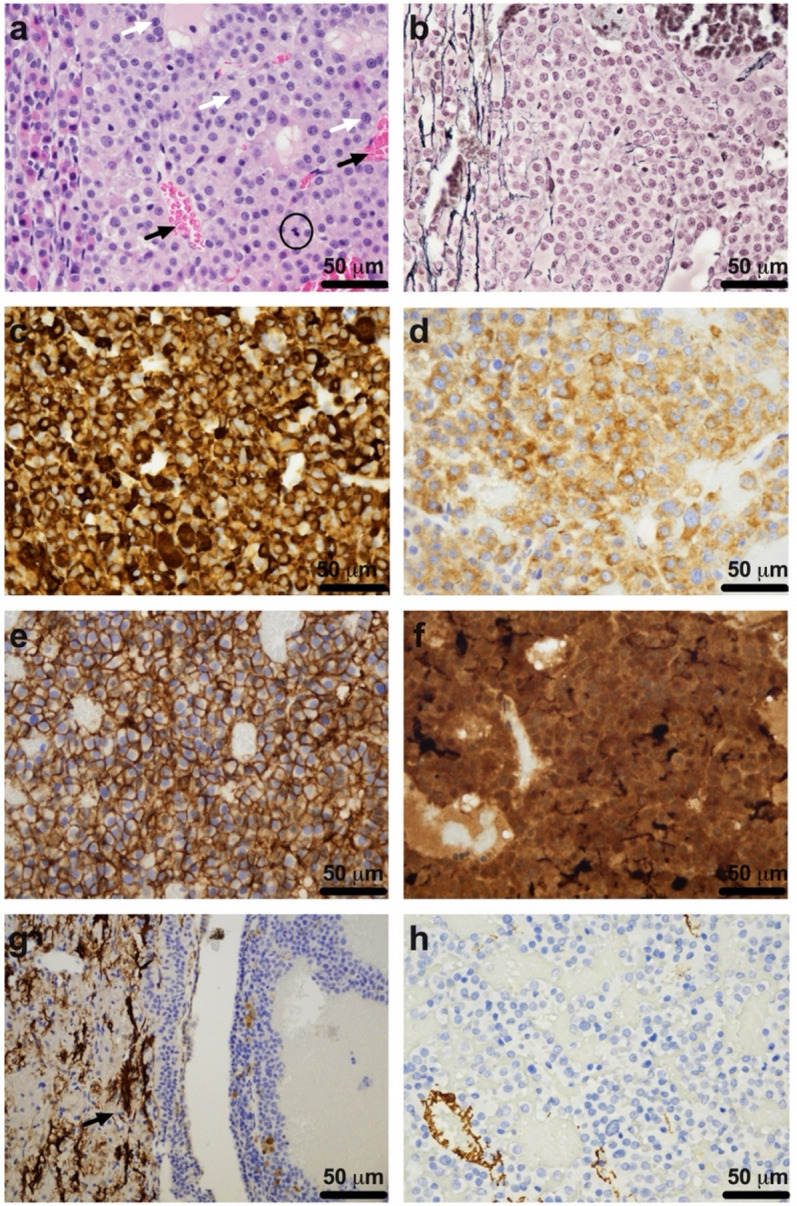
Representative tumor tissue images demonstrating the diagnostic histology and immunohistochemistry. Upon routine H&E staining **(a)**, sellar region tumors had stromal capillary networks (indicated by black arrows), monomorphic tumor cells with eosinophilic cytoplasm and round to oval nuclei with salt-and-pepper chromatin distribution (indicated by white arrows), along with evidence of mitotic activity (circled in black). Normal adenohypophysis with a mixed population of cells (left side of image) is shown adjacent to tumor tissue (right side of image). **(b)** Reticulin staining shows normal adenohypophysis within intact acinar architecture (left side of image) adjacent to tumor tissue (right side of image), with loss of reticulin-positive acinar architecture. Sellar tumors were immunoreactive for **(c)** chromogranin A, **(d)** synaptophysin, **(e)** CD56/NCAM, and **(f)** S100, but not for **(g)** GFAP (astrocytic reactivity at tumor margins indicated by black arrow) or **(h)** SMA. Routine histology (H&E, reticulin) services were provided by the University of Alberta Laboratory Medicine and Pathology Core. Immunohistochemistry services were provided by the University of Pennsylvania Comparative Pathology Core. Image scale bars indicate either 50 μm.

Sellar tumors consistently lost the reticulin-positive acinar architecture of normal adenohypophysis and displayed stromal capillary networks (Fig. 3a, b). Tumor cells were predominantly monomorphic, with an eosinophilic cytoplasm, round to oval nuclei with a salt-and-pepper chromatin distribution, and evidence of mitotic activity (Fig. 3a). Along with the tumor location, this initial work up suggested that our samples were pituitary NETs. Pituitary NETs are typically richly vascularized, display total or partial loss of the reticulin-positive acinar architecture, and exhibit characteristic salt-and-pepper chromatin distribution^36,39^. The consistent location and lack of clear “zellballen” architecture suggested that sellar tumors were not paragangliomas^40^.

#### Immunohistochemistry

Sellar tumor samples were consistently immunoreactive for neuroendocrine markers chromogranin A (Fig. 3c), synaptophysin (Fig. 3d), CD56/NCAM (Fig. 3e), and S100 (Fig. 3f), further supporting a pituitary NET classification^36,41,42^. They were not immunoreactive for GFAP (Fig. 3g) or SMA (Fig. 3h), although, as expected, there was evidence of GFAP reactivity at the tumor margins- suggestive of reactive astrogliosis^41^. Importantly, the morphological aspect and lack of immunoreactivity for GFAP and SMA within the tumor itself helps rule out gliomas, meningiomas, or smooth muscle tumors as differential diagnoses^41,42^. Additionally, while paragangliomas exhibit S100 immunoreactivity, they typically have a distinct sustentacular expression, which was not observed here (Fig. 3f)^40^.

#### Immunofluorescence

**Fig. 4:**
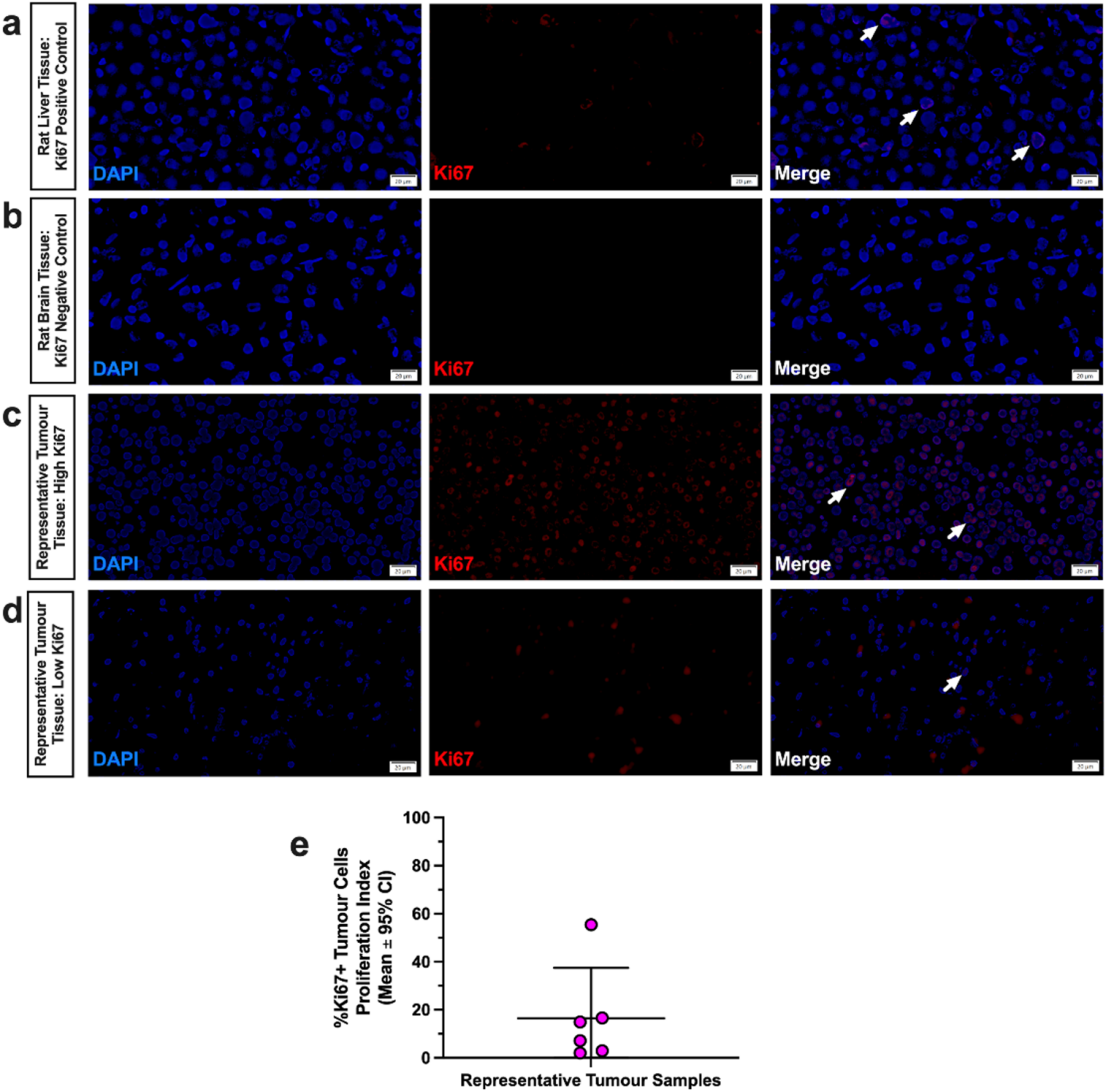
Representative immunofluorescence images of **(a)** rat liver tissue, **(b)** rat cerebellar brain tissue, and **(c**,** d)** sellar region tumor samples stained with Ki67 (a proliferation marker) and DAPI. White arrows indicate Ki67 positive cells. The average proliferation index (% of Ki67-positive cells relative to DAPI) of sellar region tumor samples are shown in **(e)**. As expected, rat liver tissue **(a)** displayed low rates of Ki67 positivity (positive control), and rat cerebellar tissue **(b)** displayed no evidence of Ki67 positivity (negative control). All sellar tumors examined displayed evidence of Ki67 positivity **(e)**; some had high proliferation rates, characteristic of grade 3 (Ki67 proliferation index > 20%) neuroendocrine carcinomas **(c)**, while others had lower proliferation rates, characteristic of grade 1 (< 3%) or grade 2 (< 20%) neuroendocrine tumors **(d)**. Image scale bars indicate 20 μm.

Of the sellar tumor samples stained with Ki67 (*n* = 6; Fig. 4a-e), two had a Ki67 proliferation index of < 3% (Fig. 4d, e). The other four samples had a Ki67 staining index of > 3%, with two samples with a Ki67 index value of > 10%, and one sample exhibiting a Ki67 index value of > 20% (Fig. 4c, e). The 2022 World Health Organization Endocrine and Neuroendocrine Tumor Guidelines classifies pituitary tumors as either pituitary NETs, or metastatic pituitary NETs, the latter of which are rare in humans (~ 0.2% of cases), and are characterized by metastases in the brain, spinal cord, or other distant tissues^43,44^. While most pituitary NETs are benign, some exhibit aggressive behaviour (e.g., such as immature PIT-1 lineage tumors, Crooke cell tumors, etc.), with rapid growth and radiologically invasive characteristics (e.g., “tumors with malignant potential without metastasis”)^44,45^. There is currently no agreed upon criteria or grading system to predict or characterize whether a pituitary NET exhibits aggressive behaviour^44^; however, pituitary NETs with a Ki67 index of ≥ 3 are proposed to be aggressive (e.g., invasive and highly proliferative)^44^, and those with a Ki67 index ≥ 10 are often considered to have high malignant potential^43–45^. Based on this criteria, four out of the six representative sellar tumor samples assessed here would be characterized as aggressive pituitary NETs.

#### Ultrastructural evaluation

**Fig. 5:**
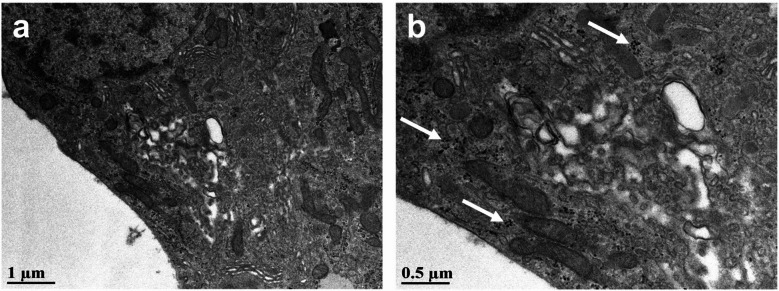
Representative ultrastructural images of a sellar region neuroendocrine tumor cell **(a)**. At a higher magnification **(b)**, small electron dense granules are visible (indicated by white arrows). These granules are not associated with rough endoplasmic reticulum, but they are smaller than typical for the secretory granules characteristic of neuroendocrine tumors, which are usually 100–400 nm in diameter.

Given their neuroendocrine origins, pituitary NETs can have a secretory capacity. Accordingly, under ultrastructural examination, secretory pituitary NET cells will often display hormonal secretory granules^16,46^. These secretory granules are electron dense and are typically ~ 100–400 nm in diameter, depending on their contents^16,46^. When examining our sellar tumor samples using TEM, tumor cells displayed small electron-dense granules that were not associated with rough endoplasmic reticulum (Fig. 5a, b). While these may be secretory granules, especially given chromogranin A immunoreactivity in other formalin-fixed paraffin embedded samples^47^, they were smaller than that typically observed in the literature^16,46^, at ~ 25–50 nm in diameter. Depending on their degree of differentiation and malignancy, pituitary NETs will not always maintain secretory capacity; alternatively, tumor cells may undergo necrotic changes that could account for our ultrastructural observations (e.g., organelle breakdown, lysosomal autolysis, protein aggregation, cellular calcification)^48^. Therefore, based on the present evidence alone, we cannot conclude whether sellar tumors in aged SHRs had any secretory capacity; further study using additional methods are required.

## Discussion

Here, we demonstrate that SHRs have a high incidence of aggressive pituitary NETs as they age. Representative sellar tumor samples displayed immunoreactivity for pituitary NET markers, such as chromogranin A, synaptophysin, S100, and CD56/NCAM^36,41,42^. Routine neuropathological stains (e.g., H&E, reticulin) supported this conclusion, revealing tumor morphology characteristic of pituitary NETs, such as stromal capillary networks and a salt-and-pepper chromatin distribution^36,39^. Further, the lack of sellar tumor immunoreactivity for GFAP or SMA ruled out a glial, meningeal, or vascular origin^41,42^. Consistent with their large sizes, most sellar tumors had a Ki67 index > 3%, indicating aggressive proliferation. The incidence rate we document here is likely an underestimate, as it was based on gross morphological evidence alone. We did not examine the pituitary region of all sixty SHRs using light microscopy to detect more subtle lesions. Importantly, this is an age-related effect, as our experiments in younger SHRs did not reveal any evidence of spontaneous intracranial tumors^32^. While various studies have documented a high incidence of pituitary NETs with age in other rat strains, especially Wistar rats, these rates are often based on microscopic observations, conducted at an older age (e.g., 24 + months)^13,16,17^. Our observations did not simply arise due to a tumorigenic environment or diet, as we have aged Sprague-Dawley rats within the same conditions without similar consequences^49^.

Notably, SHRs are derived from the Wistar Kyoto inbred rat strain, which in turn originate from the Wistar outbred rat strain^31^. Wistar rats and related strains have a known predisposition to lactotroph pituitary NETs (e.g., PIT1 lineage)^8,11,15,16,19^, though these tumors appear to be less aggressive than those observed in our study. For instance, a high incidence of lactotroph pituitary NETs was observed in an aged hybrid Wistar rat strain, but most were detectable only microscopically, and grossly visible tumors were ~ 65–70% smaller than those observed here in comparably aged SHRs^16^. More consistent with our findings, a previous study reported a pituitary NET incidence as high as 70% in an obese hypertensive rat strain (SHRs cross-bred with Koletsky rats) based on microscopic evaluation^18^. The majority of these tumors were large (> 3 mm in diameter) lactotroph pituitary NETs, developing by 18–24 months of age and following an autosomal dominant inheritance pattern^18^. Given these findings, and the pronounced age-related increase in serum prolactin levels known to occur in though these tumors appear to be less aggressive thanSHRs^50,50,52^, it is likely that their pituitary NETs are also of the lactotroph morphofunctional subtype, though direct confirmation is needed.

It is possible that the elevated risk for aggressive pituitary NETs in SHRs is associated with low expression of JunD, a transcription factor involved in inflammation and oxidative stress pathways, which is known to be deficient in SHRs due to a promoter polymorphism^29,53^. Reduced JunD levels may disrupt its interaction with the tumor suppressor protein menin, impairing menin’s crucial tumor-suppressive function in neuroendocrine tissues^54,55^, and perhaps leading to the development of aggressive pituitary NETs over time. Patients and rodents with MEN1 mutations also have disrupted menin function, leading to more aggressive pituitary NETs that are frequently of a lactotroph morphofunctional subtype^56,57^. Other proteins with low expression in SHRs, such as the cell cycle inhibitor p27^58^, are implicated in MEN syndromes^21^. A germline mutation affecting p27 is carried by both the MENX rat and humans with MEN4, and tumors develop slower with age when MENX rats are haploinsufficient^20,22^. Together, this evidence suggests a possible genetic predisposition towards aggressive lactotroph pituitary NETs in SHRs as they age, but this must be examined further.

Pituitary NETs are the third most common intracranial tumor diagnosis in patients, making up ~ 15–20% of all documented intracranial tumor cases, which in turn constitute ~ 1–2% of all cancers^59,60^. Yet paradoxically, based on autopsy and incidental neuroimaging findings, pituitary NETs are estimated to occur in ~ 10–20% of the general population^59^. This discrepancy is due to the fact that more often than not, pituitary NETs remain undetected, with only 1 out of 1000 cases resulting in clinically significant health issues that prompt diagnosis^61^. The recorded rate of clinically significant cases also may be an underestimate, given that many of the potential consequences of pituitary NETs are common with age (e.g., hypertension, diabetes, infertility, decreased libido, osteoporosis), and such issues do not typically warrant neuroimaging as part of standard diagnostic practice^62,63^. As such, the risk profile and causal factors which drive the development of pituitary NETs, and their transition to aggressive growth and invasive behaviour are not well understood outside of familial genetic syndromes, such as MEN^59,64^.

Currently, there are 35 + genes that are known to play a role in pituitary NET formation, and as it stands, current rodent models only capitulate ~ 35% of these genes^5^. Unlike the predominantly indolent behavior of pituitary NETs in humans^43^, the proliferative nature and rapid growth of these tumors in aged SHRs highlights the potential of using this rodent model to study the progression of pituitary NETs from benign to malignant or aggressive behaviour. Aggressive pituitary NETs in humans are challenging to treat, often therapy-resistant and prone to recurrence with increased morbidity and mortality^43,45^. Therefore, improving translational models of primary pituitary NET formation is a valuable exercise^56^, but the role of aged SHRs in this context remains to be seen upon further mechanistic exploration. At the very least, our longitudinal tumor observations demonstrate that caution and enhanced monitoring are warranted while aging SHRs for cardiovascular research (among other disciplines) to ensure their welfare.

This study has several important limitations. First, we did not age female SHRs in our colony. Therefore, potential sex differences in SHR tumor incidence and growth should be explored in the future, especially given that female rats of other strains are known to develop spontaneous pituitary NETs at higher rates compared to males^8–11,13,15–17,19^. Importantly, in both humans and rodents, sex-bias in pituitary tumors is highly subtype‐dependent: prolactinomas and corticotroph tumors occur more frequently in reproductive‐age females, whereas non-functioning or growth hormone-secreting tumors often show no bias or slight male predominance^8,65–67^. Given the known age-related rise in serum prolactin levels in SHRs of both sexes^50–52^, it is plausible that our large, aggressive tumors are of the lactotroph (PIT1⁺) subtype and would be more prevalent in females. However, without hormonal assays or immunostaining for lineage markers (e.g., prolactin, ACTH, PIT1, TPIT, SF1)^44,45^ and sex‐stratified analyses, we can only speculate that female SHRs may have a higher overall incidence of pituitary NETs of a given morphofunctional subtype. Future studies should include age-matched cohorts of both sexes, longitudinal monitoring of estrous cycle and hormone levels, and comprehensive subtype classification via immunohistochemistry and transcription‐factor profiling. This will clarify sex‐ and subtype-specific pituitary NET incidence rates in SHRs and elucidate the mechanisms driving tumorigenesis.

Second, although we diligently documented the occurrence of clinical tumor signs with the assistance of animal care and veterinary staff, we did not directly monitor seizure activity or the development of neurological or behavioral deficits over time in our cohort of aged SHRs. This raises the possibility that some seizures or subtle behavioral changes were either missed or too minor to be detected through standard health monitoring. Seizure activity can be monitored via telemetry^68^, including for long term periods. Smaller tumors in some animals may have gone undetected if they were not easily visible upon extraction (e.g., possible sellar origin for cerebellar tumors). Similarly, the potential occurrence of tumor metastasis could have been overlooked, despite a general visual inspection upon necropsy^44,45,69,70^. In patients, invasive pituitary NET behaviour is often evaluated radiologically^44,48^. While we were unable to do so here, notable brain infiltration was observed in most cases. The tumor mitotic index and p53 immunoreactivity is also often evaluated alongside Ki67 staining as a measure of pituitary NET proliferation, but Ki67 staining has recently been reported to be the more prognostically accurate method, with better inter-rater reliability^35^. Proliferative indices as grading criteria for pituitary NET classification remain subject to debate, however^35,71^. Our retrospective design and limited archival tissue prevented testing of candidate mechanisms proposed here. Future studies using fresh tissues, molecular profiling, and targeted pathway analyses will clarify aggressive NET pathogenesis in aged SHRs and solidify this model’s translational value for primary tumor development.

With increasing mandates for use of aged comorbid animals in research, our results stress the need for careful monitoring of these populations^23–25^. While aged SHRs may hold promise as a new rodent model of aggressive pituitary NET formation, it will be important to consider cumulative endpoints for animals held long-term^72^. This includes defining the anticipated progressive welfare impacts and humane intervention points with veterinary consult to allow adjustments such as increased monitoring, additional supportive care, and dietary supplementation^72^. Moving forward, enhanced surveillance (including regular health assessments and consideration of genetic predispositions) will be essential to mitigate these challenges and improve the robustness of research outcomes in studies involving aged comorbid animal models, regardless of discipline.

## Supplementary Information

Below is the link to the electronic supplementary material.

Supplementary Material 1

## Data Availability

All data are published alongside the article and can be found in Supplementary Material 1.
